# Towards Sustainable Development: How Digitalization, Technological Innovation, and Green Economic Development Interact with Each Other

**DOI:** 10.3390/ijerph191912273

**Published:** 2022-09-27

**Authors:** Wei Yang, Qiuxia Chen, Qiuqi Guo, Xiaoting Huang

**Affiliations:** 1 School of Management, Shandong University, Jinan 250100, China; 2 Yellow River National Strategic Research Institute, Shandong University, Jinan 250100, China

**Keywords:** sustainable development, digital economy, technological innovation, PVAR model, entropy method, econometrics

## Abstract

Green technological innovation is one of the endogenous drivers of green economic growth, and digitalization can promote green economic development in the form of industrial empowerment. The interactive relationship and the degree of influence between digitalization, technological innovation, and green economic development is thus an urgent issue to be addressed. Based on the panel data of 30 Chinese provinces from 2011 to 2019, we measured digitalization, technological innovation, and green economic development for the first time using the entropy method and included them in the same analytical framework by constructing a PVAR model to empirically test their interrelationship and degree of influence. Our findings suggest that: (1) There is an inertial development and self-reinforcing mechanism among the three variables. (2) The impact of digitalization on green economic development has a positive promotion effect, while the impact of technological innovation on green economic development is not significant. (3) The impact of green economic development on technological innovation has a positive promotion effect in the short term, but this effect gradually declines and tends to zero in the long term. Finally, based on the findings, several practical suggestions are made.

## 1. Introduction

With economic and social development and population growth worldwide, the conflict between development and the environment is gradually becoming intense [1]. Green economic development is one of the pivotal components of sustainable development, which focuses on the harmonious development of human society and nature [2]. Meanwhile, the promotion of green economic development has become an essential step to building a beautiful China, which is of great significance to achieving “new progress in the construction of ecological civilization” as proposed in the national 14th Five-Year Plan [3]. Digitalization is an essential engine that empowers green economic development. Digitization refers to a crucial means that deems digital knowledge and information as a key production factor, modern information networks as an important carrier, and the use of information and communication technology as an effective approach, ultimately promoting technological innovation, efficiency improvement, and economic structure optimization. Its high technological sophistication, growth, and cleanliness can provide a new path for China to achieve green economic development [2].

In recent years, digitization and related technological innovations in China have advanced promptly and taken the leading positions in some fields [4]. The basic idea of digitization is to make it possible to manufacture products and provide services, innovate in the field of technology, and replace old growth drivers with new ones through the dissemination and processing of elements supported by modern information technology in the context of market globalization and sustainable development [5]. In addition, the Internet world is gradually shifting from information orientation towards value orientation, and the concept of digitization is gaining importance. Digitization stimulates technological innovation and provides new forms and carriers for social economy and enterprises, offering a broader business scope and economic efficiency to the market [6].

In the past two decades, global digitalization has shown a rapid growth trend and has become a new engine in the world’s economic recovery [4]. The pandemic has ravaged the world since 2020, leading to a significant decline in the global economy and social development, but digitalization has alleviated and stabilized the economic downward trend of many countries to some extent. During COVID-19, digitization played a fundamental role in combating the pandemic, supporting public health management, maintaining social order, and achieving the resumption of work and schooling [7,8]. Digitalization can energize traditional economic activities and shows wide application and great growth potential with the advantages of network and data space compared to the traditional physical economy that relies on physical space. Digitalization has become one of the most dynamic and innovative economic and social forms and core growth poles of the national economy. It is essentially a special economic form of trading goods and services through virtualization [5]. Digitization makes access to information easy, makes interaction active, and is very cost-effective [9]. It plays an essential role in the decision-making, production, marketing, trade, distribution, and service activities of enterprises and is deeply integrated with the specific shape of the real economy [10]. It has become an important driving force and a new dynamic for green economic development [11]. It is thus speculated that the green economic development of economic entities in the post-pandemic era will urgently require digitalization.

Previous studies argued that digitalization can have a profound impact on the quality of economic development, which is illustrated in the following aspects: First, digitalization can realize the centralized integration and efficient utilization of production factors. Digitization development can make elements of data through physical carriers to achieve networked sharing, intensive integration, and efficient utilization of technology, labor, capital, and resource elements, which ultimately leads to exponential improvement of economic and social operation efficiency [12,13]. Second, digitalization has changed the traditional production and consumption mode. On the one hand, digitalization has realized significant changes in objects of labor, means of production, and labor force, and promoted the exponential growth of productivity [14,15]. To be specific, at the level of objects of labor, digitalization has transformed objects of labor from traditional materials to massive data elements; at the level of means of production, digitalization has transformed means of production from machine systems to physical information systems [16]; and at the level of the labor force, digitalization has transformed the labor force from industrial workers to digital labor [17]. On the other hand, digitalization can take the economic value network as a link to continuously break the barriers of information asymmetry in the industry, enhance the flow speed of information elements, and give birth to the platform economy, sharing economy, virtual space consumption, artificial intelligence plus, Internet plus, and a series of new economic forms as well as business models to accelerate the advent of the “pro-consumer” era. Digitalization can effectively improve the comprehensive governance capacity of government departments [18]. China is at the critical stage of starting a new journey of building a great modern socialist country in all respects. As there are many problems such as increasingly acute environmental pollution and resource depletion globally, the decreasing domestic demographic dividend, the increasing downward pressure on the economy, and the middle-income trap [19], the government is facing unprecedented challenges. While digitization can reveal deep connections that are difficult to show under traditional governance methods by promoting government data sharing and openness and implementing digital governance, which can improve government management effectiveness. As a whole, the existing literature generally agrees that digitization can be a major driver of economic and social transformation and development [11].

Moreover, digitalization is a dynamic rather than a static form and carrier, so it can lead to more significant benefits and provide a stronger boost to other directions of development [20]. However, there is a paucity of research that specifically explores the links and impacts of digitalization, technological innovation, and green economic development through quantitative research [21]. In the new economic and social development stage, the green value of digitalization will certainly be emphasized, and its role in sustainable development will be highlighted. Therefore, how to seize the major development opportunities brought by the new round of technological revolution to provide strong momentum for China’s green economic development through digitalization is one of the crucial issues that needs urgent attention in China’s economic development in the 14th Five-Year Plan period. Thus, exploring the relationship between digitalization, technological innovation, and green economic development and the practical path is not only conducive to seeking innovative directions at the theoretical level but also to making comprehensive plans at the industrial practice level.

Based on the above analysis and the literature review, the entropy method was used in this study to measure digitization, technological innovation, and green economic development for the first time using panel data of 30 Chinese provinces from 2011 to 2019, and a PVAR model was constructed to explore the interaction between digitization and technological innovation as well as green economic development and the degree of influence.

The main marginal contributions of this study are reflected in three aspects: First, digitalization, technological innovation, and green economic development were placed in the same research framework for the first time, and an in-depth econometric analysis of the impact of digitalization and technological innovation on green economic development was conducted to enrich the quantitative research in the field of digitalization, technological innovation, and green economic development. Second, more comprehensive indicators were used to measure the research variable, including green, innovation, coordination, sharing, and openness to thoroughly evaluate the level of green economic development, and the entropy method was used to effectively measure digitalization, technological innovation, and green economic development. Third, dynamic panel models, GMM tests, and impulse response analysis were used to investigate the potential linkage between digitalization, technological innovation, and green economic development. The different promoting effects of digitalization and technological innovation on green economic development and their corresponding characteristics at different development stages are revealed, consequently providing references for the formulation of relevant strategies and industrial practices.

The paper is divided into six sections. Section 1 introduces the background of the study, the source of the selected topic, the significance, the research questions, and the theoretical contributions. Section 2 is the review of the relevant literature. Section 3 contains the mechanism and theoretical analysis, and the research framework is proposed accordingly. Section 4 presents the methodology used, the variables, and the data selection. Section 5 elaborates on each process of empirical test and analyzes the results. Section 6 summarizes the main findings of this study, discusses the conclusions obtained, and makes practical suggestions for digitalization, technological innovation, and green economic development based on the empirical analysis.

## 2. Literature Review 

China’s digital economy will reach CNY 45.5 trillion in 2021, with year-over-year increase of 16.2%, accounting for 39.8% of the GDP in that year [22]. The position of the digital economy in the national economy has become more solid and supportive. As digitalization becomes the main engine of the development of the digital economy, digital transformation has been an inevitable option for traditional industries to comply with the new situation of the times and economic development. Digitalization or digital transformation is the integration of digital technology into business processes, and digital transformation has a profound impact on value creation, delivery, and acquisition in many industries [23].

At the industry level, the use of digital technologies provides opportunities to integrate products and services across functional, organizational, and geographic boundaries [24]. Digital technologies have accelerated the process of industrial transformation and led to significant changes in many industries [25,26]. With the help of digital empowerment, companies have an endogenous drive to overturn traditions, thus further driving technological change across industries [25]. The concept of “Industry 4.0” or “smart factory” [27] was further introduced to leverage technologies such as cloud computing and the Internet of Everything on a larger scale to optimize each process in the production management chain [28]. Leveraging big data management allows stakeholders in the supply chain system to share information, facilitate the flow of factors, reduce redundant links, and improve productivity [29,30,31]. Moreover, it limits the potential for abuse. Digitalization has revolutionized the way industry works [32]. Digital platforms have created a new method of operation for companies and organizations in the “business ecosystem” [33], leading to the constant iteration and growth of industry value networks [34].

Digitalization and technological innovation have a complex and dialectical relationship, and the full picture cannot be seen from the industry level alone. Therefore, some scholars have analyzed it from the perspective of internal innovation. In the context of the booming digital economy, the digital transformation of enterprises has become an inevitable trend [35]. Having a timely, continuous, granular, and complete information structure is the hallmark of the digital transformation of enterprises [36]. In turn, digital proliferation and embedding are considered opportunities for enterprise innovation and transformation [37] the powerful penetration capacity of which makes them widely used in production activities and business management activities in various industries and boosts digital transformation and structural optimization of traditional enterprises [38]. Hoffman’s theorem argues that technological innovation affects changes in industrial structure from changes in production costs, prices, and resource allocation, so technological innovation is one of the key paths to realizing structural transformation of factor endowments and optimization and upgrading of industry structure. Digital technology can complement other production and operation management technologies to reconfigure and integrate various factor resources, including production and organizational methods, triggering production paradigm improvements and industrial linkage effects and promoting structural optimization of production sectors [39].

When companies carry out internal reforms and digital transformation, they need to rely on new skills to innovate, learn, and adapt to evolving digital technology requirements, and digitalization can change the originally compiled knowledge of production and innovation activities [14]. The application of digital technology accelerates the clustering and flow of knowledge [40] and gradually blurs the boundaries of innovation stages, making digital product and service innovation characterized by rapid iterations and upgrades, while it can also improve the dynamic capabilities of innovative processes such as dual capabilities, reorganization capabilities, and digital technology adaptation capabilities of companies [41]; drive breakthrough innovation; and promote sustainable transformation and development of industries [42]. Digital technology enables unlimited data replication and sharing and instant interconnection and has unique advantages in reducing data processing costs and transaction costs as well as precisely allocating resources, which can enhance enterprise productivity by reducing expenditure and improving efficiency [43]. Digital technology also accelerates capital deepening through the accumulation of ICT capital, increases capital support for corporate innovation and R&D investment to boost productivity progress, and ultimately enhances corporate productivity [43,44].

From a macro point of view, digitalization is playing an increasingly essential role in many economic entities around the world. Some scholars considered the structure of the digital economy in Asia and found that digital development can lead to business and social change as a means of triggering digitally relevant consumer demand and digital governance [45], and stimulant policies made by the government together with digital entrepreneurship can improve business processes and enrich the business landscape, both of which ultimately drive growth changes in the digital economy [46]. Based on panel data from G7 economic entities over the period 1990 to 2017, the study found that in terms of digitalization, firm-funded R&D expenditures, revenue, and financial risk, there was a significant increase in technological innovation in the G7 economies [47]. Digital global trade in services is part of the digitization of the economy and trade. Scholars analyzed the development trend and influence factors of digital global trade in services based on panel data of digital global trade in services trade for 33 countries from 2005 to 2020, and the results showed that digital infrastructure, human capital, and technological innovation capability have a significant impact on countries’ digital deliverable trade, among which the level of technological innovation has the most significant impact, and the role of digital trade in services that is played in sustainable economic and social development among countries is increasingly prominent [48]. Digitalization is a crucial condition for the transformation and innovation of financial institutions [49], while financial institutions accelerate the transformation and development of digital finance through technological innovation, and digital finance improves the competitiveness of national capital markets, reduces the cost of searching and transacting financial resources, thus reducing the cost of financing, which helps to promote the steady and constant transformation of technological innovation into productivity [50,51,52,53,54]. 

Digitalization had fundamentally changed traditional business models and patterns [55,56,57], social linkages, and interactions, consequently increasing the centralization of national markets [58]. Specifically, economic systems became widely shared, circular, and sustainable. Moreover, technological innovation has significant spillover effects [59,60] that significantly empower economic growth, which in turn ensures the country’s green economic development [61].

## 3. Theoretical Analysis

A review of the relevant academic literature reveals that the impact of digitalization on green economic development is multi-level, multi-dimensional, and compound [62]. Emerging technologies such as the Internet and e-commerce can form an economic environment with economies of scale, economies of scope, and long-tail effects. Based on that, improving the equilibrium level of the economy and enhancing the efficiency of economic activities can be realized by better-matched supply and demand and a better price mechanism at the micro level. At the macro level, the new input factors, new resource allocation methods, and new total factor productivity will jointly promote green economic development. 

**Figure 1:**
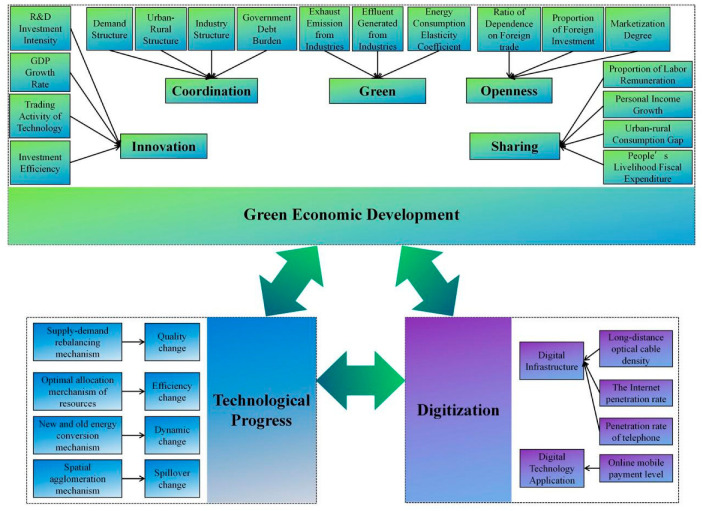
Theoretical framework (source: made by authors).

As technological innovation is an endogenous driver of economic growth [63,64], it plays an important role in promoting the replacement of traditional drivers with new ones, upgrading economic structure, and improving productivity and resource allocation efficiency as well as social vertical mobility. Green economic development can also provide strong support for promoting digitalization and technological innovation. However, the previous literature does not assess the interaction relationship and influence degree between digitalization, technological innovation, and green economic development. This study combines existing studies [65,66,67,68] and theoretical analysis to provide a framework for this purpose (Figure 1).

## 4. Research Method, Variable Selection, and Data Sources

### 4.1. Research Method

To explore the dynamic influence relationship between the digital economy, technological innovation, and green economic development, a panel autoregression (PVAR) model was constructed with data from 30 Chinese provinces (cities, autonomous regions, and municipalities directly under the central government) from 2011–2019 (excluding Tibet, Hong Kong, Macao, and Taiwan), which allows all variables to be endogenous and reflects the dynamic relationship among variables. The research model is as follows.

(1) $$Y_{it}=\alpha_{0}+\sum_{j=1}^{n}\alpha_{j}Y_{i,t-j}+\beta_{i}+\gamma_{i}+\varepsilon_{it}$$

*Y_it_* = (lndata, lntin, lneco) is a three-dimensional column vector; lndata indicates the level of digitalization; lntin indicates the level of technological innovation; lneco indicates green economic development; ln indicates the variables taken as logarithms; *α*_0_ is the intercept term; *j* is the lag order; *a_j_* is the parameter matrix of lag order *j*; *β_i_* is the individual fixed effect; *γ_i_* is the individual time-point effect; and *ε_it_* is the random disturbance term.

### 4.2. Variable Selection

We chose digitalization, technological innovation, and green economic development as the main variables.

#### 4.2.1. Digitalization (lndata)

As for digitalization, the current literature has not yet reached a unified measurement index. In order to ensure the scientificity of digitalization index measurement, most scholars construct comprehensive indicators to indirectly measure the development level of digitalization. According to previous studies, digital infrastructure and digital technology applications are representative and comprehensive evaluation systems for measuring digitalization. Therefore, we drew on the existing literature [22,69,70] to construct a comprehensive indicator system from two perspectives: digital infrastructure and digital technology application. Relying on the development of the Internet, digitalization is widely used in e-commerce and digital finance. Therefore, digital infrastructure was measured with three indicators: long-distance optical cable density, Internet penetration rate, and penetration rate of telephone. Digital technology application had two indicators: online mobile payment level and the digitization degree of digital finance. The decomposition and weights of the indicators are shown in Table 1.

#### 4.2.2. Technological Innovation (Lntin)

The technological innovation index mainly focuses on the input and output of technological innovation. Based on previous studies [71], the full-time equivalent of research and experimental development (R&D) personnel and internal expenditure of R&D funds were used as human and capital inputs, respectively, and the number of patent applications received and the technology turnover in the technology market were used to reflect the technological innovation activity and technological innovation output. In this study, MATLAB software and the entropy method were used to measure the weight of each index, and the specific index decomposition and the weight of each index are shown in Table 2.

#### 4.2.3. Green Economic Development (Lneco)

It has been widely agreed that green economic development is the goal of all economies in the world. Although a consensus has been reached in the academic community that total factor productivity is an important factor for the sustainability of economic growth, limited by the volatility of measurement and the single dimensionality, it is obvious that it cannot meet the research needs as a lone evaluation indicator of green economic development. Therefore, an increasing number of scholars measure the level of green economic development through a multidimensional index system. However, a consensus on measurement indicators of green economic development has not yet been reached, and this work draws on Reference [72] to measure green economic development from five dimensions: innovation, coordination, green, openness, and sharing. The MATLAB software and entropy method were used to measure the weight of each indicator, and the specific information is shown in Table 3.

### 4.3. Data Sources

The research sample of this study contains 30 provinces (cities, autonomous regions, and municipalities directly under the Central Government) in China from 2011–2019 (excluding Tibet, Hong Kong, Macao, and Taiwan). The data of technology innovation-related indicators were obtained from the China Science and Technology Statistical Yearbook 2011–2020, the data of green economic development-related indicators were obtained from China Statistical Yearbook 2011–2020 and the China Regional Economic Statistical Yearbook 2011–2020; and the digitalization-related indicators were obtained from China Communication Yearbook 2011–2020 and China Internet Development Status Statistical Report.

## 5. Data Analysis and Results

### 5.1. Stationarity Test and Optimal Lag Order Selection

Although panel data mitigate the non-stationarity of data to some extent, individual variables may still have trend and intercept problems, resulting in pseudo-regression phenomena. To ensure the robustness of the research results, four types of tests were used in this study, LLC, IPS, ADF, and PP, to conduct unit root tests on the variables: digitalization (lndata), technological innovation (lntin), and green economic development (lneco). Results can be seen in Table 4, and digitalization (lndata) passed the 1% significance level in all four tests. Technological innovation (lntin) passed the 1% significance level in both LLC and PP tests and passed the 5% significance level in the ADF test. However, green economic development (lneco) did not pass the IPS test. It passed the 1% significance level in both LLC and PP tests but did not pass the IPS test and ADF test. In short, all three variables are smooth variables.

Before conducting PVAR model estimation, in order to ensure the validity of the estimation, the optimal lag order of the model should be determined. In this study, we used the PVAR2 program package of STATA 13.0 to select the optimal lag order with AIC, BIC, and HQIC. Results can be seen in Table 5 for the three detection criteria, and the first-order lag order is optimal, so the PVAR model of one-phase lag is the most appropriate.

### 5.2. Co-Integration Test Results

Based on the data smoothness test, the Pedroni co-integration test was used to verify whether there was a long-run equilibrium relationship among the variables. The results of the Pedroni co-integration test shown from Table 6 reject the original hypothesis of no co-integration relationship among the variables at the 1% significance level. Thus, we can conclude that there is a long-term stable equilibrium relationship among the three variables of digitalization, technological innovation, and green economic development.

### 5.3. Analysis of Granger Test Results

To further analyze the short-term dynamic influence effect and logical relationship between digitalization, technological innovation, and green economic development, the Granger causality test was conducted for each variable. As can be seen in Table 7, technological innovation and digitalization exhibit Granger causality at a 10% significance level.

Technological innovation and green economic development exhibit Granger causality along with technological innovation and digitalization, while green economic development shows the one-way Granger causality of digitalization. The 5% significance level was chosen for the test, indicating that the joint effect of the variables can dynamically predict the explanatory variables in the short term. In brief, there is a strong Granger causality among the three variables, but the specific causality has to be further tested by tools such as GMM estimation and impulse response function.

### 5.4. GMM Estimation Results

To examine the influence of the lagged term of each variable, we used STATA software to conduct GMM estimation of the PVAR model constructed by the three variables of digitalization, technological innovation, and green economic development. Conclusions can be drawn from Table 8: First, the estimation of technological innovation, digitalization, and green economic development with one lagged period are together all positive and pass the 1% significance level, indicating that the development of three variables characterizes inertial development characteristics and self-reinforcing mechanisms. Second, when technological innovation (lntin) is the explained variable, the estimation of digitalization with one lagged period is positive and passes the 10% significance level, indicating that digitalization has a positive impact on technological innovation. The estimation of green economic development with technological innovation is negative and does not pass the significance test, indicating that the impact of green economic development on technological innovation is not strong in the short term. Third, when digitalization (lndata) is regarded as the explained variable, the estimation of technological innovation is positive with one lagged period and passes the 10% significance test, suggesting that technological innovation has a positive contribution to the development of digitalization because technological innovation is the basic support and prerequisite for digitalization. The estimation of green economic development with digitalization with one lagged period is positive, and it does not pass the significance test, suggesting that the impact of green economic development on digitalization is not strong. Lastly, when green economic development is considered the explained variable, the estimation of technological innovation is negative with one lagged period and does not pass the significance test, showing that the impact of technological innovation on green economic development is not strong. The estimation of digitalization with green economic development is positive, and it passes the 1% significance test, showing that the impact of digitalization on green economic development is strong. This is due to fact that the development of digitalization has given rise to a large number of business models in the economy and society, and new business models and new transaction scenarios rely heavily on the development and application of digitalization, so digitalization can positively promote green economic development. Though the GMM estimation results can reflect the correlation between variables in a more macroscopic way, the specific dynamic transmission process and the degree of response of each variable in the face of shocks of other variables need to be further analyzed. 

### 5.5. Analysis of Impulse Response Results

**Figure 2:**
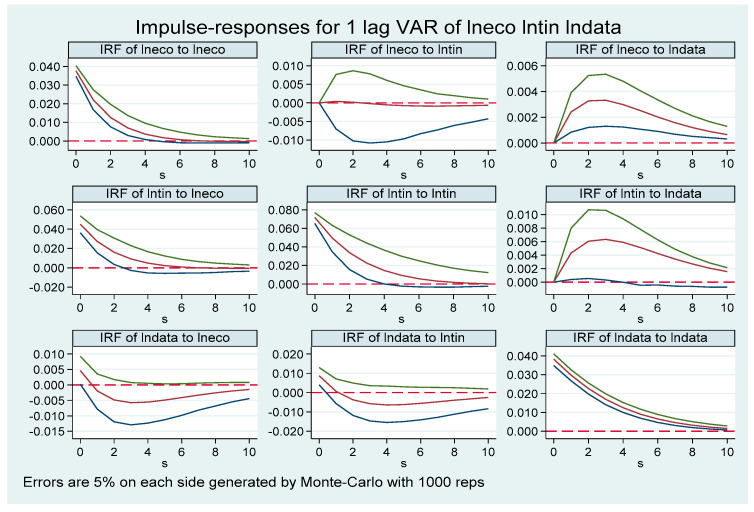
Pulse response results of digitalization, technological innovation, and green economic development.

To further characterize the specific dynamic interaction process and influence effects between digitalization, technological innovation, and green economic development, impulse response figures were obtained with 10 lagged periods based on 1000 Monte Carlo simulations at the 95% confidence interval (Figure 2). Impulse response refers to the impact of a variable on itself as well as other variables when the random disturbance term is subjected to a shock of one standard deviation, which can visually reflect the dynamic time-lagged interaction relationship among the variables. From the impulse response diagram, conclusions can be drawn: First, each variable responds positively to the shock from itself and reaches its maximum in the current period, and then this response gradually declines until it disappears, which again confirms the inertia characteristics of technological innovation, digitalization, and green economic development in the development practice. Second, when facing one standard deviation shock of technological innovation, the response of green economic development is zero in the current period, followed by a weak negative response, reaching a maximum in the sixth period, then gradually decreases and approaching zero. When facing one standard deviation shock of digitalization, the response of technological innovation is zero in the current period, then gradually increasing, reaching a maximum in the third period, then gradually decreases and approaching zero, which shows an overall positive response. Third, when subjected to a standard deviation shock of digitalization, the response of technological innovation is zero in the current period, gradually increases, and reaches the maximum in the third period. Then, the response decreases and eventually approaches zero. The long-term dynamic response trend shows an inverted “U” pattern. When confronting a standard deviation shock of green economic development, technological innovation responds positively in the current period and reaches the maximum, reacts to zero in the sixth period, and gradually tends to zero in the long term. Finally, when confronting a standard deviation shock of green economic development, digitalization reacts positively in the current period, gradually shows a negative reaction from the first period, and tends to zero in the long term. When facing a standard deviation shock of technological innovation, digitalization reacts positively in the current period, gradually shows a negative reaction from the first period, and tends to zero in the long term. The overall response strength is relatively flat.

### 5.6. Variance Decomposition

On the basis of impulse response analysis, the proportional contribution of each variable shock to the fluctuation of endogenous variables was measured by variance decomposition to further verify the degree of impact among variables. The results can be seen in Table 9: (1) The three variables of technological innovation (lntin), digitalization (lndata), and green economic development (lneco) contribute much more to themselves than the other two variables, indicating that all three variables have self-reinforcing mechanisms in development. Specifically, the contribution of technological innovation is as high as 100% in the first period of the self-shock, gradually decreases with time and reaches 97.8% in the 10th period, and gradually tends to zero in the long run. The contribution rate of digitalization is as high as 93.8% in the first period of its shock and experiences slight change but still reaches 88.5% in the 10th period. The contribution rate of green economic development is as high as 71.8% in the first period of its shock and declines steadily with time but still reaches 70.0% in the 10th period. (2) For technological innovation, in addition to its own impact, digitalization has a greater contribution rate compared to green economic development. Specifically, when facing one standard deviation shock of digitalization, the response of technological innovation is zero in the first period and increases over time, reaching 1.6% in the 10th period. When facing one standard deviation shock of green economic development, the overall response of technological innovation is relatively weak, only 0.6% in the 10th period. (3) For digitalization, besides its own contribution rate, the contribution rate of technological innovation shows a trend from small to large, with the contribution rate of 6.2% in the first period, then rising to 11.3% in the 10th period. The contribution rate of green economic development is 0 in the first period and 0.2% in the 10th period so the contribution rate appears weak. (4) For green economic development, apart from its own larger impact, technological innovation has a greater contribution rate compared to digitalization. To be specific, the contribution rate of technological innovation to green economic development shows an inverted U-shaped change pattern, which is 28.2% in the first period and reaches a peak in the second period then gradually decreases and finally remains at 27.8%. Moreover, the contribution rate of digitalization to green economic development shows a change from small to large; it is zero in the first period then gradually increases and reaches 2.2% in the 10th period.

## 6. Conclusions and Discussion

### 6.1. Conclusions

The development of the green economy is of great significance to the realization of sustainable development goals of economic society. Thus, this study analyzed the dynamic impact between digitalization, technological innovation, and green economic development based on Chinese provincial panel data from 2011–2019, and a PVAR model was constructed. The following conclusions can be drawn: (1) There is an inertial development and self-reinforcing mechanism among digitalization, technological innovation, and green economic development both in the short and long term. (2) From the perspective of green economic development, the impact of technological innovation is not significant, while the impact of digitalization has a positive promotion effect in the short or long term. Digitalization has a greater long-term impact on green economic development compared to technological innovation. (3) From the perspective of technological innovation, green economic development has a positive promotion effect in the short term, but this effect gradually decreases and tends to zero in the long term. The impact of digitalization has a positive promotion effect in the short or long term. Digitalization has a greater long-term impact on technological innovation compared with green economic development. (4) From the perspective of digitalization, the impact of green economic development on digitalization is positive in the current period and then becomes negative in the long run, and the effect of this negative impact shows an inverted U-shape. Furthermore, the impact of technological innovation on digitalization is positive in the current period and then turns negative, and the impact of this negative effect also shows an inverted U-shaped trend. Compared to green economic development, technological innovation has greater influence effect on digitalization.

### 6.2. Discussion

#### 6.2.1. Theoretical Implications

The primary marginal contributions of this paper are reflected in three aspects: First, this work places digitization, technological innovation, and green economic development in the same research framework and provides an in-depth econometric assessment of the impact of digitization and technological innovation on green economic development, but a majority of previous studies have used qualitative analysis to study digitization and green economic development [67]. This study utilized the quantitative analysis approach to enrich quantitative research in the field of digitalization, technological innovation, and green economic development.

Second, a more comprehensive set of indicators was used to measure the variables studied. For instance, we adopted the five dimensions of green, innovation, coordination, sharing, and openness to thoroughly measure the level of green economic development. We also used the entropy method to effectively measure digitalization, technological innovation, and green economic development. Research on green economic development to date remains focused on a single indicator or simple accumulation of several indicators. On the other hand, the indicator system merely covers a specific industry, such as ecological agriculture or tourism industry [73], but there is no sufficient three-dimensional and multi-dimensional indicator system to measure the concept of green economic development [16,74]. Though both eco-agriculture and tourism industries have been regarded as smokeless industries and green economies, this is not sufficient for measuring green economic development comprehensively.

Finally, the study adopted dynamic panel models, GMM tests, and impulse response analysis to investigate the potential linkages between digitalization, technological innovation, and green economic development. The empirical findings suggest that digitalization can effectively promote green economic development in China. Past studies have utilized panel data from the Yangtze River Economic Belt in China [68]. However, this study took a step forward in this area by utilizing panel data from 30 provinces, and more convincing results were obtained. In addition, this study reveals the different promotion effects of digitalization and technological innovation on green economic development at different development stages and their corresponding merits, which provide suggestions for the related strategic formulation and industrial practices.

#### 6.2.2. Practical Implications

This study provides useful managerial implications for the transformation and upgrading of the economy to green development. Firstly, rational allocation of production factors can be guided by policy to achieve cost reduction and efficiency promotion, encourage enterprises to carry out technological innovation, and complete digital transformation through technical support. Enterprises can adapt to the new situation of the digital market and further promote the development of the digital economy. Next, it is pivotal to create a good environment for the development of the digital economy, release the demand for infrastructure construction of the digital economy, and optimize existing digital resources with 5G technology, the Internet of Things, and other new technologies, consequently making joint efforts to promote green economic development. Furthermore, the industrial Internet platform should be constructed swiftly and promote the application of technologies such as big data, the Internet, and artificial intelligence in the industry to advance the interconnectivity of information infrastructure and the degree of openness and sharing of digital resources. We should try to avoid the phenomenon of data islands and enrich the application scenarios of the digital economy to create a good digital environment. The hard environment and soft environment will pave the way for digitalization to be transformed into productivity. Eventually, the endogenous factor of technological innovation will provide an impetus for green economic development.

Secondly, it is worth noting that digital infrastructure and digital transformation enterprise are still at the early stage of the life cycle [75], so it is necessary to consider the investment in digital infrastructure, the maturity of technology, and the degree of integration with industry, as well as the certain crowding-out effect of technological innovation of the industry. Traditional digital industrial enterprises which lack technical support are especially common in China. Therefore, it is proposed that industrial enterprises should establish digital infrastructure in an orderly manner according to the digital development law and give full play to the technology accumulation effect in the early implementation process to complete the digital infrastructure empowerment, which will promote the regional green economy development.

### 6.3. Limitations and Future Prospects

This study examines the dynamic interaction between digitization, technological innovation, and green economic development, but there are still some limitations. To start with, this work is based on the provincial level and does not consider the spatial correlation of green economic development in each province. In the future, the spatial interaction between digitalization, technological innovation, and green economic development can be explored using spatial econometric models based on the spatial spillover effect of green economic development. Next, 30 provinces in mainland China were selected for analysis, but the digitalization, technological innovation, and green economic development may vary greatly among provinces. In the future, specific provinces, such as Zhejiang Province, the leading green economic development area, or Shanghai, the enterprises of which are more advanced in green development transformation, can be selected for a more in-depth analysis by using the case study method. The case study approach combined with empirical research can provide deeper analysis and conclusions. Finally, this study investigated the interrelationship between digitalization, technological innovation, and green economic development, and more relevant factors can be incorporated into the study to obtain more comprehensive conclusions by combining theoretical analysis and realistic situations.

## Figures and Tables

**Table 1:** Index selection and weight for digitalization.

First Level Indicator	Second Level Indicators	Third Level Indicators	Indicator Weight
Digitalization	Digital infrastructure	Long-distance optical cable density (+)	0.631
		Internet penetration rate (+)	
		Telephone penetration rate (+)	
	Digital technology application	Online mobile payment level (+)	0.369

Note. “+”is positive indicator. The same is as follows. (source: made and calculated by authors using the entropy method).

**Table 2:** Index selection and weight for technological innovation.

First Level Indicator	Second Level Indicators	Third Level Indicators	Indicator Weight
Technological innovation	Input for technological innovation	Internal expenditure of R&D funds (+)	0.390
		The full-time equivalent of research and experimental development (R&D) personnel (+)	
	Output for technological innovation	Number of patent applications received (+)	0.610
		Technology turnover in the technology market (+)	

(Source: made and calculated by authors using the entropy method).

**Table 3:** Index selection and weight for green economic development.

First Level Indicator	Second Level Indicator	Third Level Indicator	Indicator Weight
Green economic development	Innovation	GDP growth rate (+)	0.401
		R&D investment intensity (+)	
		Investment efficiency (−)	
		Trading activity of technology (+)	
	Coordination	Demand structure (+)	0.139
		Urban–rural structure (+)	
		Industry structure (+)	
		Government debt burden (−)	
	Green	Energy consumption elasticity coefficient (−)	0.031
		Effluent generated from industries (−)	
		Exhaust emission from industries (−)	
	Openness	Ratio of dependence on foreign trade (+)	0.347
		Proportion of foreign investment (+)	
		Marketization degree (+)	
	Sharing	Proportion of labor remuneration (+)	0.083
		Elasticity of personal income growth (+)	
		Urban–rural consumption gap (−)	
		Proportion of people’s livelihood fiscal expenditure (+)	

Note. “+” is positive indicator; “−” is negative indicator. (Source: made and calculated by authors using the entropy method).

**Table 4:** Unit root test results.

	Lndata	Lntin	Lneco
LLC test	−50.715 ***	−3.976 ***	−6.748 ***
IPS test	−25.599 ***	1.202	0.849
ADF test	390.715 ***	78.070 **	0.387
PP test	581.764 ***	152.224 ***	115.095 ***

Note. *** means passing 1% significance test. ** means passing 5% significance test.

**Table 5:** Test results of optimal lag order selection.

lag	AIC	BIC	HQIC
1	−8.89673 *	−7.3188 *	−8.25883 *
2	−8.58226	−6.66648	−7.80549
3	−7.04367	−4.69537	−6.08963

Note. * means the optimal lag order.

**Table 6:** Co-integration test results.

Program	Estimation	*p*-Value
Modified Phillips–Perron t	6.164	0.00
Phillips–Perron t	−3.117	0.00
Augmented Dickey–Fuller t	−56.711	0.00

**Table 7:** Granger causality test results.

Program	Causality	Chi-Squared	Degrees of Freedom	*p*-Value
Technological innovation	Digitalization is not the cause.	3.513	1	0.061
	Green economic development is not the cause.	0.153	1	0.695
	All variables are not the cause.	3.857	2	0.145
Digitalization	Technological innovation is not the cause.	3.314	1	0.069
	Green economic development is not the cause.	0.152	1	0.696
	All variables are not the cause.	12.418	2	0.002
Green economic development	Technological innovation is not the cause.	0.000	1	0.975
	Digitalization is not the cause.	6.430	1	0.011
	All variables are not the cause.	11.843	2	0.003

**Table 8:** GMM estimation results.

Variables	Lntin		Lndata		Lneco	
	Estimation	Z Value	Estimation	Z Value	Estimation	Z Value
L1. lntin	0.679 ***	5.640	0.085 *	−1.820	−0.002	−0.030
L1. lndata	0.113 *	1.870	0.778 ***	27.760	0.063 **	2.540
L1. lneco	−0.101	−0.390	0.047	−0.390	0.581 ***	4.130

Note. *** means passing 1% significance test. ** means passing 5% significance test. * means passing 10% significance test.

**Table 9:** Variance decomposition results.

Period	Lntin			Lndata			Lneco		
	Lntin	Lndata	Lneco	Lntin	Lndata	Lneco	Lntin	Lndata	Lneco
1	1.000	0.000	0.000	0.062	0.938	0.000	0.282	0.000	0.718
2	0.997	0.002	0.001	0.040	0.959	0.001	0.285	0.002	0.712
3	0.993	0.005	0.002	0.043	0.955	0.002	0.284	0.007	0.709
4	0.989	0.008	0.004	0.058	0.940	0.002	0.282	0.011	0.706
5	0.985	0.010	0.005	0.074	0.924	0.002	0.280	0.015	0.704
6	0.982	0.012	0.005	0.087	0.910	0.002	0.279	0.018	0.703
7	0.981	0.014	0.006	0.098	0.900	0.002	0.279	0.020	0.702
8	0.980	0.015	0.006	0.105	0.893	0.002	0.278	0.021	0.701
9	0.979	0.015	0.006	0.110	0.888	0.002	0.278	0.021	0.700
10	0.978	0.016	0.006	0.113	0.885	0.002	0.278	0.022	0.700

Unit: %.

## Data Availability

Not applicable.
